# Efficacy of combined lung and heart treatment method for yang deficiency with water overflowing type Chronic Obstructive Pulmonary Disease: A prospective cohort study

**DOI:** 10.12669/pjms.41.12.12503

**Published:** 2025-12

**Authors:** Zhonghui Zhou, Zhengyu Xie, Xier Chen, Yali Yu

**Affiliations:** 1 Zhonghui Zhou, Department of Respiratory Medicine, Ningbo Municipal Hospital of Traditional Chinese Medicine (TCM), Affiliated Hospital of Zhejiang Chinese Medical University, Ningbo, Zhejiang, 315012, China; 2 Zhengyu Xie, Department of The First School of Clinical Medicine, Zhejiang Chinese Medical University, Hangzhou, Zhejiang, 310053, China; 3 Xier Chen, Department of Respiratory Medicine, Ningbo Municipal Hospital of Traditional Chinese Medicine (TCM), Affiliated Hospital of Zhejiang Chinese Medical University, Ningbo, Zhejiang, 315012, China; 4 Yali Yu, Department of Respiratory Medicine, Ningbo Municipal Hospital of Traditional Chinese Medicine (TCM), Affiliated Hospital of Zhejiang Chinese Medical University, Ningbo, Zhejiang, 315012, China

**Keywords:** Combined lung and heart treatment method, Cohort study, Chronic obstructive pulmonary disease, Traditional Chinese Medicine, Yang deficiency with water overflowing

## Abstract

**Objective:**

To evaluate the efficacy of combined lung and heart treatment (CLHT) for Yang deficiency with water overflowing type chronic obstructive pulmonary disease (COPD).

**Methodology:**

This is a prospective cohort study. A total of 92 patients with this TCM subtype COPD admitted to Ningbo Hospital of Traditional Chinese Medicine Affiliated to Zhejiang Chinese Medical University were randomly assigned to treatment (n = 46) and control (n = 46) groups from December 2020 to February 2023. The control group received standard Global Initiative for Chronic Obstructive Lung Disease guideline-based inhaled therapy. The treatment group received oral Traditional Chinese Medicine (TCM) decoction for twelve weeks. TCM symptom scores, COPD Assessment Test (CAT) scores, forced expiratory volume in one second (FEV_1_), forced vital capacity (FVC), FEV_1_/FVC, serum concentrations of interleukin-6 (IL-6) and -8, annual acute exacerbation frequency and adverse events were used for treatment evaluation.

**Results:**

Baseline characteristics were comparable (*P* > 0.05). Post-treatment, both groups showed significant reductions in TCM symptom scores, CAT scores, IL-6 and -8 levels and annual acute exacerbation frequency (*P* < 0.05), with greater improvements in the treatment group (*P* < 0.05). FEV_1_, FVC and FEV_1_/FVC showed significant improvement only in the treatment group (*P* < 0.05), with FEV_1_ and FEV_1_/FVC higher in the treatment group than the control (*P* < 0.05). Furthermore, the treatment group achieved a higher total efficacy rate (90 % vs. 64. *1%; χ^2^ = 7.258; P < 0.05) without drug-related adverse events*.

**Conclusion:**

The CLHT method improved the clinical efficacy of Yang deficiency with water overflowing type COPD treatment, alleviated clinical symptoms, enhanced patients’ quality of life, improved pulmonary function and reduced annual acute exacerbation frequency.

## INTRODUCTION

Chronic obstructive pulmonary disease (COPD), the third leading cause of death worldwide,^1^ is a common chronic airway disease involving the respiratory system. Recent epidemiological data from China indicated that COPD has a prevalence of 13.7% among individuals aged ≥ 40 years.^2^ Global projection models estimate a 23% increase in COPD cases among adults aged ≥ 25 years from 2020 to 2050, affecting nearly 600 million patients worldwide by 2050.^3^ Despite receiving standard therapy, many COPD patients continue to experience persistent symptoms and recurrent acute exacerbations.^4^ Chronic hypoxia and recurrent acute exacerbations in patients with COPD contribute to the progressive development of chronic cor pulmonale, ultimately resulting in heart failure and other adverse outcomes.^5^ Therefore, there is an urgent need for complementary and alternative therapies to address this critical challenge.

COPD corresponds to the Traditional Chinese Medicine (TCM) syndromes Fei Zhang (lung distension). The TCM pathology of Fei Zhang typically progresses from lung Qi deficiency to heart-kidney Yang deficiency. The pathogenesis of TCM mirrors the clinical trajectory of COPD progression toward pulmonary hypertension and/or cor pulmonale. A series of studies previously demonstrated that combination TCM-Western therapy significantly alleviated clinical symptoms, enhanced quality of life and exercise tolerance, improved pulmonary function and reduced acute exacerbation frequency in patients with COPD through syndrome differentiation-guided interventions.^6^-^9^ Yang deficiency with water overflowing, a TCM syndrome observed in advanced COPD, manifests as dyspnea, edema and cold intolerance as a result of impaired cardiopulmonary Yang Qi and fluid metabolism.

The primary objective of the combined lung and heart treatment (CLHT) method is to prevent or control the adverse consequences of lung diseases that affect the heart. Clinical practice has demonstrated favorable therapeutic outcomes in the treatment of COPD using this method.^10^ This study aimed to investigate whether integrating TCM with standard therapy improves patient outcomes.

## METHODOLOGY

This prospective clinical cohort study included 92 patients diagnosed with Yang deficiency with water overflowing type COPD who were treated in the Respiratory Department of Ningbo Municipal Hospital of TCM Affiliated Hospital of Zhejiang Chinese Medical University between December 2020 and February 2023. Participants were randomized into treatment (n = 46) and control (n = 46) groups using a random number table.

### Ethical Approval:

The protocol for this study was approved by the ethics committee of Ningbo Municipal Hospital of TCM Affiliated to Zhejiang Chinese Medical University (approval no. AF/SG-01/01/20201215001; dated December 8, 2020). All participants provided written informed consent prior to treatment.

### Inclusion criteria:

- Patients met the diagnostic criteria for COPD.^11^
- COPD classified as lung distension and Yang deficiency with water overflowing type based on TCM.^12^
- Age between 40 and 80 years.
- Patients did not participate in other clinical studies, received treatment voluntarily and signed an informed consent form.

### Exclusion criteria:

- Tuberculosis, pulmonary fungal infections, lung cancer or other primary lung diseases.
- Patients with severe liver, kidney or hematopoietic system diseases.
- Pregnant or lactating women.
- Patients with mental illness.
- Patients with poor compliance.

### Dropout criteria:

- Patients who were requested to withdraw from the study or failed to complete the treatment and follow-up periods as planned.
- Patients who completed the entire treatment cycle but whose primary data were incomplete and treatment efficacy was not determined.
- Patients who did not meet the inclusion criteria during the study period.

### Therapeutic protocol:

Patients in the control group received inhaled drugs according to the Global Initiative for Chronic Obstructive Lung Disease (GOLD), including long-acting muscarinic antagonist (LAMA), long-acting beta-agonist (LABA)/LAMA, inhaled corticosteroid (ICS)/LABA or ICS/LABA/LAMA.^9^ The treatment group (CLHT method) received inhaled protocols and a TCM decoction, the ingredients of which were as follows: Pseudostellariae Radix 15 g, Astragalus 30 g, Rhodiola rosea 10 g, Salvia miltiorrhiza 15 g, Polygonum cuspidatum 15 g, Lumbricus 9 g, Epimedium 12 g, Fagopyrum dibotrys 30 g, Morus alba 12 g, Tetrastigmatis Hemsleyani Radix 6 g, Polyporus 10 g, Poria 15 g, Alisma orientalis 12 g, Cassia twig 9 g, Atractylodes macrocephala 10 g and Descurainiae Semen 10 g. Each patient ingested half a dose twice daily for 4 weeks (1 course). The efficacy was evaluated after three courses.

### Outcome measures:

### TCM syndrome scores:

The following symptoms were observed: cough, expectoration, chest tightness, shortness of breath, palpitations, limb edema, fatigue, appetite, chills and cold limbs. Scores (0, 1, 2 or 3) were assigned according to symptom severity and changes in TCM syndrome scores were documented before and after treatment.

### Life quality:

CAT scores^13^ were used to measure patients’ quality of life before and after treatment.

### Pulmonary function:

Changes in FEV_1_, FVC and FEV_1_/FVC before and after treatment were compared.

### Inflammatory markers:

Serum concentrations of IL-6 and -8 were determined by immunofluorescence assay before and after treatment.

### Annual acute exacerbation frequency:

The number of acute exacerbations during the year prior to treatment were recorded. A follow-up call was conducted one year after treatment to document the number of acute exacerbations in the year following treatment.

### Adverse reactions:

Drug-related adverse reactions were monitored in the two groups of patients during the treatment course.

### Clinical efficacy:

TCM symptom scores were calculated using the nimodipine method to determine clinical efficacy. Efficacy index = (pre-treatment score - post-treatment score) / pre-treatment score × 100 The criteria for efficacy were as follows: clinical cure, efficacy index ≥ 95%; markedly effective, efficacy index ≥ 70%; effective, efficacy index ≥ 30%; and ineffective, efficacy index < 30%.^14^ Total effective rate (%) = (number of clinically cured cases + number of markedly effective cases + number of effective cases) / total number of cases × 100%.

### Statistical analyses:

Statistical analysis were performed using SPSS version 22.0 (IBM Corp., Armonk, NY, USA). All count data were tested for significant differences using the Chi-squared test, while the t-test was used for normal data and non-parametric tests were used for skewed data. Statistical significance was set at *P* < 0.05.

## RESULTS

**Fig.1:**
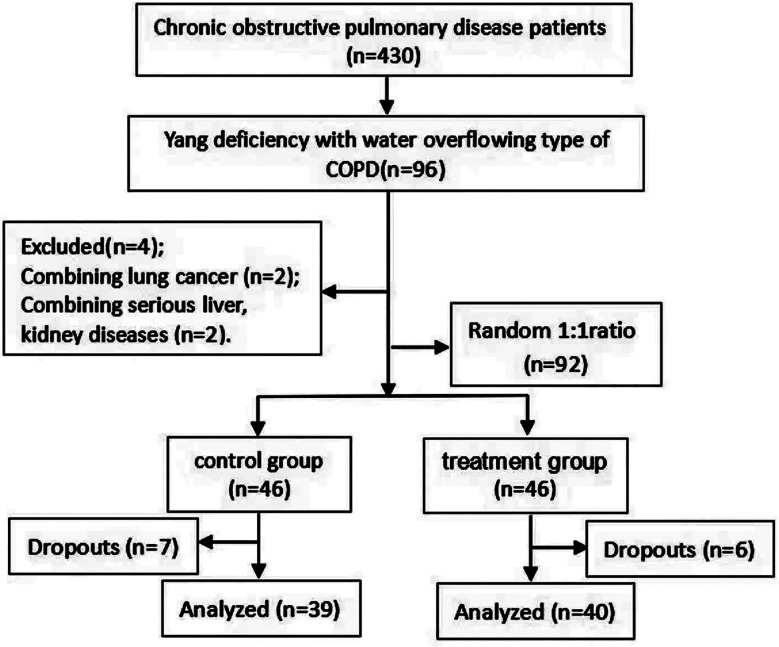
The flowchart of the study.

The screening protocol applied in this study is depicted in Fig.1. There were no significant differences in sex, age, disease duration or lung function severity between the two groups (*P* > 0.05) (Table-I). Before treatment, there were no statistically significant differences in the TCM syndrome and CAT scores (*P* > 0.05); however, after treatment, the TCM syndrome and CAT scores of both groups were reduced (*P* < 0.05), with a greater reduction in the treatment group (Table-II).

**Table-I:** Comparison of baseline participant characteristics.

Characteristics	Control group (n = 39)	Treatment group (n = 40)	χ^2^/t - value	P - value
Sex (M/F), n	32/7	30/10	0.579	0.446
Age (mean ± SD), years	68.90 ± 8.15	67.10 ± 7.21	-1.039	0.302
Course of disease (mean ± SD), years	10.87 ± 7.03	10.91 ± 8.55	0.020	0.984
Severity of lung function			0.462	0.794
Grade II	11	14		
Grade III	22	21		
Grade IV	6	5		

**Table-II:** Comparison of TCM syndrome and CAT scores between the two groups (mean ± standard deviation).

Item	Time	Control group (n = 39)	Treatment group (n = 40)
TCM syndrome scores	Before treatment	14.49 ± 5.12	14.55 ± 2.24
	After treatment	10.08 ± 3.34^a^	5.88 ± 3.01^ab^
CAT scores	Before treatment	20.74 ± 5.12	20.75 ± 3.74
	After treatment	15.65 ± 5.23^a^	10.40 ± 3.47^ab^

***Notes:***

a : compared with before treatment, P < 0.05,

b : compared with control group, P < 0.05.

Before treatment, there were no statistically significant differences in FEV_1_, FVC and FEV_1_/FVC (*P* > 0.05). After treatment, FEV_1_, FVC and FEV_1_/FVC significantly increased in the treatment group (*P* < 0.05), whereas there were no significant differences in the control group (*P* > 0.05). Compared with the control group, FEV_1_ and FEV_1_/FVC in the treatment group were significantly higher after treatment (*P* < 0.05), whereas FVC did not change significantly (*P* > 0.05) (Table-III). Before treatment, there were no statistically significant differences in the serum concentrations of IL-6 and -8 (*P* > 0.05); however, after treatment, the serum concentrations of IL-6 and -8 were significantly reduced in both groups (*P* < 0.05), with the difference being more pronounced in the treatment group (*P* < 0.05) (Table-III).

**Table-III:** Comparison of pulmonary function, cytokine levels and annual acute exacerbation frequency between the two groups (mean ± standard deviation).

Item	Time	Control group (n = 39)	Treatment group (n = 40)
FEV_1_ (L)	Before treatment	1.08 ± 0.47	1.04 ± 0.36
	After treatment	1.05 ± 0.45	1.26 ± 0.40^ab^
FVC (L)	Before treatment	2.13 ± 0.73	2.13 ± 0.66
	After treatment	2.11 ± 0.74	2.32 ± 0.66^a^
FEV_1_/FVC (%)	Before treatment	50.28 ± 10.03	49.81 ± 9.15
	After treatment	49.59 ± 10.94	54.85 ± 10.78^ab^
IL-6 (ng/L)	Before treatment	19.44 ± 15.20	18.70 ± 14.81
	After treatment	11.22 ± 7.15^a^	6.38 ± 4.88^ab^
IL-8 (ng/L)	Before treatment	40.27 ± 29.33	39.19 ± 24.88
	After treatment	19.55 ± 8.19^a^	14.43 ± 7.46^ab^
Annual acute exacerbation frequency	Before treatment	1.67 ± 0.90	1.58 ± 0.93
	After treatment	0.95 ± 0.60^a^	0.50 ± 0.60^ab^

***Notes:***

a : compared with before treatment, P < 0.05,

b : compared with control group, P < 0.05.

The total efficacy in the treatment group was 90% (36/40), whereas in the control group it was 64.1% (25/39; χ^2^ = 7.258; *P* < 0.05) (Table-IV). Before treatment, there were no statistically significant differences in the annual acute exacerbation frequency (*P* > 0.05); however, one year after treatment, the annual acute exacerbation frequency in both groups was significantly lower than before treatment (*P* < 0.05), with the annual acute exacerbation frequency in the treatment group significantly lower than that in the control group (*P* < 0.05) (Table-III). No drug-related adverse reactions occurred in either group during the course of treatment.

**Table-IV:** Results of effectiveness evaluation after treatment in the two groups (n, %).

Item	Control group (n = 39)	Treatment group (n = 40)	χ^2^- value	P - value
Clinical cured	0 (0)	0 (0)	7.25	0.006
Markedly effective	4(10.3)	16(40)		
Effective	21(53.8)	20(50)		
Ineffective	14(35.9)	4(10)		

## DISCUSSION

The result of this study demonstrated the superior therapeutic efficacy of the CLHT method when used in combination with standard therapy in patients with Yang deficiency with water overflowing type COPD. The treatment group exhibited a higher overall response rate and greater reduction in TCM symptom scores, CAT scores and serum concentrations of IL-6 and -8 and concurrent improvements in FEV_1_ and FEV_1_/FVC at 12-weeks post-treatment. At the 12-month post-treatment follow-up, the treatment group exhibited a significantly lower annual frequency of acute exacerbations than the control group.

COPD is characterized by cardinal symptoms of cough, sputum, and dyspnea, progressing to palpitations and edema in later stages. A clear inverse correlation exists between the intensity of these symptoms and the patient’s health-related quality of life.^15^ The results of this study demonstrated that the TCM decoction significantly improved both TCM symptom and CAT scores, indicating that the CLHT method effectively alleviated the clinical symptoms of COPD and enhanced quality of life. FEV_1_ and FEV_1_/FVC serve as key indicators of airflow limitations, with FEV_1_ being particularly crucial for determining the severity of COPD. The findings of this study revealed significant improvements in FEV_1_ and FEV_1_/FVC with the TCM decoction compared with the control group, suggesting that the CLHT method may improve pulmonary function. Compared to baseline, no improvement in lung function was observed in the control group, which may be attributed to suboptimal adherence to inhalation therapy or improper inhaler technique.^16^,^17^ Current uncertainty regarding the effects of TCM on COPD pulmonary function^6^,^18^ may stem from heterogeneity in syndrome differentiation, baseline treatment regimens and whether initial pulmonary function tests were conducted during acute exacerbations.

COPD is a chronic airway inflammatory disorder, the pathogenesis of which involves multiple interleukins. IL-6 and -8 exacerbate inflammation by inducing the infiltration of neutrophils, monocytes and eosinophils into the airway mucosa while simultaneously stimulating extracellular matrix components to promote fibroblast proliferation and airway remodeling.^19^,^20^ IL-6 levels are also correlated with excessive airway mucus secretion in patients with COPD.^21^ Patients with elevated levels of inflammatory mediators, such as IL-6 and -8, exhibit aggravated clinical manifestations, as systemic inflammation directly impairs pulmonary function and diminishes quality of life.^22^ These pathophysiological correlations have led to the widespread adoption of IL-6 and -8 as critical biomarkers for monitoring therapeutic efficacy in clinical studies. The results of this study demonstrated that the CLHT method, when used in combination with inhaled medications, significantly reduced serum IL-6 and -8 levels in patients with Yang deficiency with water overflowing type COPD compared with controls. This suggests that TCM attenuates airway inflammation through cytokine modulation, thereby decreasing airway resistance, improving pulmonary function and ultimately alleviating respiratory symptoms while enhancing exercise capacity. Acute exacerbation is the primary mortality risk and a major disease burden in COPD.^23^ The results of this study showed significantly fewer annual acute exacerbations in the TCM intervention group, indicating that the CLHT method may reduce the risk of acute exacerbation in these patients.

### Strength of the Study:

Our study provides multi-dimensional evidence that integrating TCM with standard therapy offers superior benefits for COPD patients. These findings underscore the clinical value of the CLHT method as a complementary and alternative approach for patients who remain symptomatic and experience frequent exacerbations despite conventional treatment.

### Limitations:

This was a single-center cohort study with a moderate sample size and cardiac parameters (e.g., echocardiography and B-type natriuretic peptide levels) were not systematically evaluated. Therefore, large-sample, multicenter, prospective cohort studies are needed to confirm the effects of TCM on cardiovascular and pulmonary function. Owing to the distinctive nature of the TCM decoction intervention, blinding was not feasible in this study, which may have introduced potential expectation bias.

## CONCLUSIONS

TCM using the CLHT method improved the clinical efficacy of treatment for Yang deficiency with water overflowing type COPD, improved clinical symptoms, quality of life and pulmonary function and reduced the annual acute exacerbation frequency. The therapeutic mechanism behind this decoction likely involves the suppression of IL-6 and -8 levels.

### Authors’ Contribution:

**ZZ:** Conceived and designed the study.

**ZZ, XC and YY:** Collected the data.

**ZZ and ZX:** Performed statistical analysis of data, manuscript writing and responsible for the accuracy and integrity of the study.

**ZZ, ZX:** Contributed equally to this work and are listed as co-first authors.

All authors have read and approved the final manuscript.
